# A Case of Primary Squamous Cell Carcinoma of the Liver Arising in a Solitary Cyst

**DOI:** 10.1155/1992/32474

**Published:** 1992

**Authors:** O. Nieweg, M. J. H. Slooff, J. Grond

**Affiliations:** 1 Department of Surgery and Department of Pathology University Hospital Groningen The Netherlands; 2 University Hospital Groningen Department of Surgical Oncology Oostersingel 59 Groningen 9734 EZ The Netherlands

## Abstract

A case of primary squamous cell carcinoma in a pre-existing hepatic cyst is presented. A review of the literature suggests that this rare type of liver tumor tends to arise from solitary, nonparasitic cysts, lined with squamous epithelium. Effective therapy is not available, the prognosis is grave.


*HPB Surgery*, 1992, Vol. 5, pp. 203–208
Reprints available directly from the publisher
Photocopying permitted by license only

© 1992 Harwood Academic Publishers GmbH
Printed in the United Kingdom

### CASE REPORT
### A CASE OF PRIMARY SQUAMOUS CELL CARCINOMA OF THE LIVER ARISING IN A SOLITARY CYST

O. NIEWEG, M.J.H. SLOOFF and J. GROND
*Department of Surgery and Department of Pathology, University Hospital Groningen,*

*(Received 13 August 1991)*

A case of primary squamous cell carcinoma in a pre-existing hepatic cyst is presented. A review of the literature suggests that this rare type of liver tumor tends to arise from solitary, nonparasitic cysts, lined with squamous epithelium. Effective therapy is not available, the prognosis is grave.

KEY WORDS: Squamous cell carcinoma, liver tumor

### INTRODUCTION

Histologically primary hepatic cancer is usually hepatocellular carcinoma. Intrahepatic bile duct carcinoma is less frequently found. Rare primary tumors are squamous cell carcinoma, adenocarcinoma (Willis, 1943), adenosquamous carcinoma (Barr, 1975), mucoepidermoid carcinoma (Pianzola, 1971), cystadenocarcinoma (Cruickshank, 1971) and various types of sarcoma (Iwatsuki, 1988). This report describes a patient with primary squamous cell carcinoma of the liver.

### CASE

A sixty-two year old female was referred with a tumor of the liver. Needle biopsy of the lesion in another hospital had shown that the lesion could possibly be squamous cell carcinoma.

The patient complained of pain in the right side of the abdomen of five month duration. There was weight loss of two kilograms in the previous two months. She could not tolerate fatty foods. The stools had been very lightly coloured on two occasions. The medical history was otherwise unremarkable.

Physical examination revealed yellow tinged sclerae and a visible swelling in the right upper quadrant of the abdomen. The right lobe of the liver was palpable four cm. below the costal margin and was irregular and tender.

---

Address correspondence to: O. Nieweg, M.D., Ph.D., University Hospital Groningen, Department of Surgical Oncology, Oostersingel 59, 9734 EZ Groningen, The Netherlands
Supported by the Dutch Cancer Foundation "Koningin Wilhelmina Fonds".

Hemoglobin concentration was 112 g per l, leucocyte count 12 × $10^9$ per l, platelet count 430 × $10^9$ per l and the erythrocyte sedimentation rate was 58 mm per hour. Serum electrolytes were normal and liver enzymes were raised (alkaline phosphatase 437 U per l, LDH 457 U per l, GOT 128 U per l, GPT 209 U per l, gamma glutamyl transpeptidase 317 U per l). Bilirubin and cholinesterase were within the normal range. Blood coagulation test results were normal. Serological markers for the hepatitis B virus were present but no antigen was detected. Carcinoembryonic antigen and alpha-fetoprotein were within normal limits.

An ultrasonogram showed a filling defect of 11 × 12 × 15 cm with a hypodense zone in the right lobe of the liver. A lymph node of one cm. was seen next to the coeliac axis. A CT-scan showed that the lesion extended across into segment II (Figure 1).

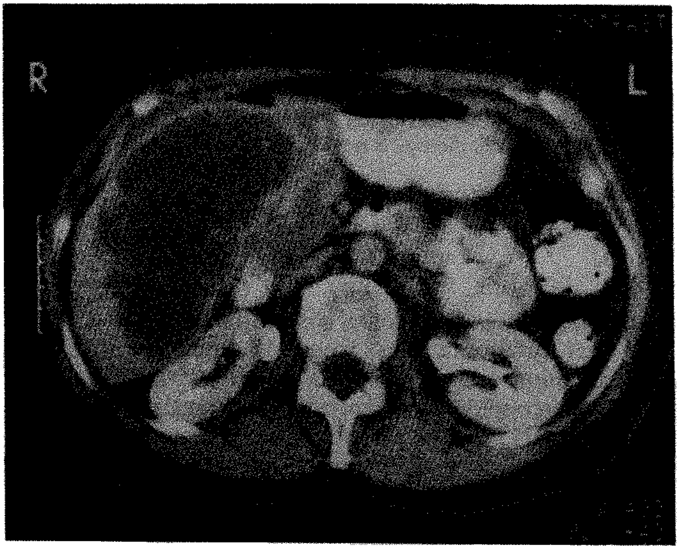

**Figure 1** Roentgen-CT scan showing a large, irregular lesion in the liver.

The possibility that the lesion represented a hepatic metastasis of a malignancy elsewhere in the body was considered. A thorough search for a primary tumor was undertaken including chest radiography, bronchoscopy, tomography of the lungs, oesophago-gastroscopy, intravenous urography, gynecological examination, barium enema, mammography, thyroid scintigraphy and ear, nose and throat screening. No primary tumor was found $^{57}$Co-bleomycin scintigraphy for tumor detection demonstrated the lesion in the liver but no abnormalities elsewhere in the body. Bone scintigraphy was normal.

Since no primary tumor elsewhere in the body was found and since no certain diagnosis was available, laparotomy was performed in order to obtain a pathologic diagnosis and to consider resection. The lesion in the right lobe of the liver was found to have invaded the stomach, colon and greater omentum rendering resection impossible. Adhesions prevented systematic examination of the remainder of the abdominal cavity. A small piece of the lesion was excised for histologic examination. It then became apparent that the lesion was a cyst with a tumorous wall, filled with slightly purulent fluid (Figure 2).

Histologic examination of fragmented biopsy material revealed necrosis, an inner layer of compact fibrous tissue containing sparse inflammatory cells, surrounded by a loose fibrous layer containing blood vessels and bile ducts. In the cyst wall widespread cords and nests of highly pleomorphic cells with abnormal mitotic figures and focal squamous differentiation were observed, characteristic of squamous cell carcinoma (Figure 3). Mucus stains were negative. No preexistent epithelial cyst lining was observed.

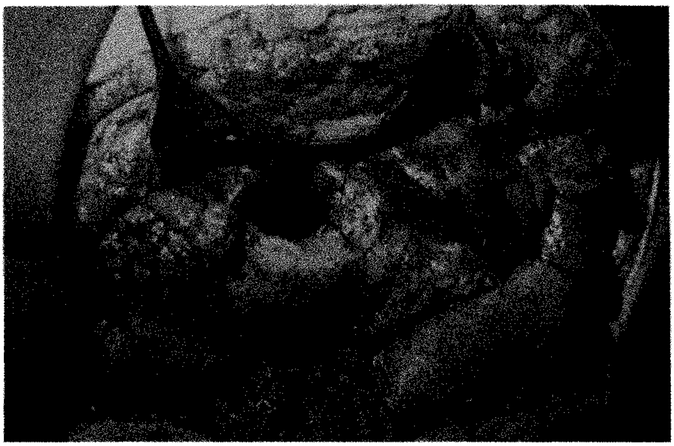

**Figure 2** A subcostal incision was made. The abdominal wall is retracted cranially. In the lower right corner the stomach is visible. The liver is seen in the middle. The biopsy revealed a cyst.

Culture of the fluid from the cyst revealed Enterococci.

Postoperatively the patient did well initially. Later on she became progressively weaker and died five months after laparotomy. Autopsy was not performed.

### DISCUSSION

We report, here, a case of primary squamous cell carcinoma of the liver. There are no other such cases on file in our hospital. The tumor is thought to have originated in a pre-existing cyst. The presence of microorganisms in the fluid within the cyst was probably caused by secondary infection.

Primary squamous cell carcinoma of the liver is an exceedingly rare type of tumor. To our knowledge thirteen cases have been described (Imai, 1934, Edmondson, 1958, Greenwood, 1972, Bloustein, 1976, Song, 1984, Gresham, 1985, Pawelczak, 1987, Iwatsuki, 1988, Lynch, 1988, Mandai, 1989). The youngest patient was 30 years of age, the oldest 78. All nine patients whose sex was reported were male.

The first case described in the literature, is consistent with malignant degeneration in a teratoma (Imai, 1934). Another case has been reported to arise in a liver with multiple intrahepatic cholesterol stones (Song, 1984). Eight cases arose in

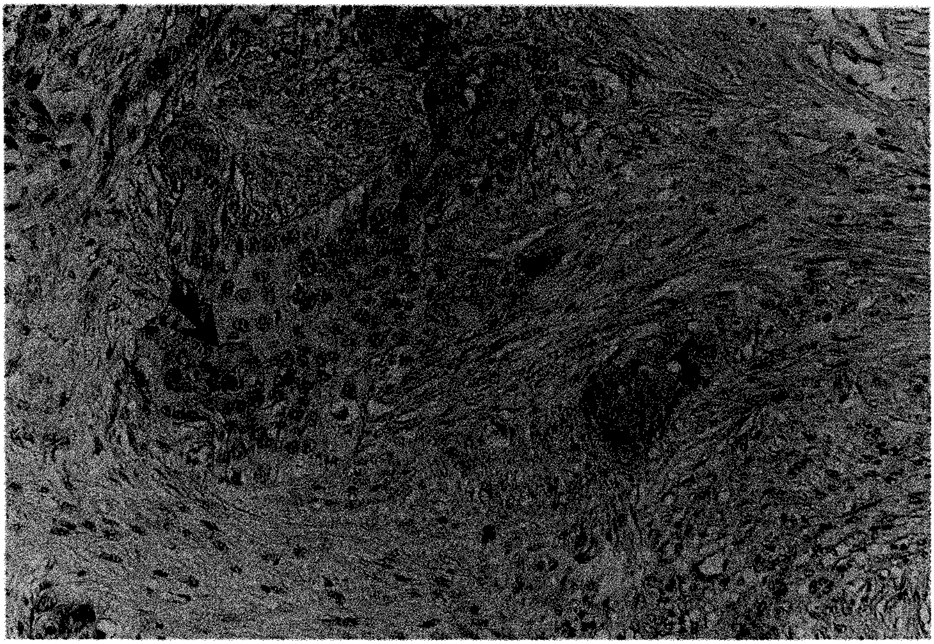

**Figure 3** High power micrograph of squamous cell carcinoma in the liver cyst wall. Arrow indicates squamous differentiation. Hematoxylin & eosin, × 140.

solitary cysts. Six of these patients were male, of the other two the sex was not mentioned. This is a remarkable sex distribution since solitary liver cysts occur in women approximately three times more frequently than in men (Flagg, 1967, Sanfelippo, 1974). These eight cysts, with subsequent carcinoma, were congenital or acquired, but not parasitic. Various types of epithelial lining can be found in these cysts (Sanfellipo, 1974). When a lining of squamous epithelium is present, dysplasia and malignant degeneration may arise in longstanding cysts (Edmondson, 1958, Greenwood, 1972, Bloustein, 1976, Gresham, 1985, Lynch, 1988). Although squamous cell carcinoma in general tends to develop central necrosis, the fact that they are largely lined by innocuous epithelium excludes the possibility that the cysts arise from necrosis in a pre-existing solid tumor. It is conceivable that a cyst enlarges because of the presence of a carcinoma and thus brings about its symptoms.

Symptoms of this type of tumor include pain and loss of weight. On examination jaundice and a palpable mass in the epigastrium are found. CT seems to be the most valuable pre-operative investigation. At laparotomy a large tumor, often within a cyst, is found. It is frequently found to be adherent to adjacent structures, making resection difficult. Metastases may be found in the lymph nodes, thus precluding a cure. Haematogenous metastases have not been explicitly described. When metastases are present resection of the primary tumor may be considered for palliation. Since squamous cell carcinoma elsewhere in the body is usually fairly sensitive, radiation therapy may be of value for palliation. The prognosis is poor, the mean survival is 6 months, the longest recorded survival was 16 months after laparotomy.

Solitary, nonparasitic cysts of the liver are rare. With the more liberal use of ultrasonography and CT the diagnosis may be established more often. Apart from the above described cases of squamous cell carcinoma a number of other types of neoplasia have been reported to arise in solitary liver cysts: hepatoma (Richmond, 1956), adenocarcinoma (Iwatsuki, 1988, Ameriks, 1972), cystadenocarcinoma (Lloyd Jones, 1974), adenosquamous carcinoma (Barr, 1975, Pianzola, 1971) or a combination of the last two (Moore, 1984). However, the risk of a malignancy arising in a solitary liver cyst is small. Therefore, we do not advocate major hepatic resections for solitary nonparasitic liver cysts when simple removal is precluded by the proximity of vital structures.

*Acknowledgement*

We wish to thank Dr P. Pizarski for his help during the preparation of the manuscript.

### *References*

Willis, R.A. (1943) Carcinoma arising in congenital cysts of the liver. *J.Pathol.Bacteriol.*, **55**, 492–495

Barr, R.J. and Hancock, D.E. (1975) Adenosquamous carcinoma of the liver. *Gastroenterology*, **69**, 1326–1330

Pianzola, L.E. and Drut, R. (1971) Mucoepidermoid carcinoma of the liver. *Am. J. Clin. Pathol.*, **56**, 758–761

Cruickshank, A.H. and Sparshott, S.M. (1971) Malignancy in natural and experimental cysts - Experiments with aflatoxin in rats and the malignant transformation of cysts in human livers. *J. Pathol.*, **104**, 185--190

Iwatsuki, S. and Starzl, T.E. (1988) Personal experience with 411 hepatic resections. *Ann. Surg.*, **208**, 421–434

Imai, T. (1934) Ein Fall von zystischem Teratom der Leber, in welchem Plattenepithelkrebs entstand. *Trans. Soc. Pathol. Jap.*, **24**, 578–580

Edmondson, H.A. (1958) Atlas of tumor pathology. Section VII-Fascicle 25. Tumors of the liver and intrahepatic bile ducts. Washington: Armed Forces Institute of Pathology, 109–111

Greenwood, N. and Orr, W. (1972) Primary squamous cell carcinoma arising in a solitary nonparasitic cyst of the liver.*J. Pathol.*, **107**, 145–148

Bloustein, P.A. and Silverberg, S.G. (1976) Squamous cell carcinoma originating in a hepatic cyst. Case report with a review of the hepatic cyst-carcinoma association. *Cancer*, **38**, 2002–2005

Song, E., Kew, M.C., Grieve, T., Isaacson, C. and Myburgh, J.A. (1984) Primary squamous cell carcinoma of the liver occurring in association with hepatolithiasis. *Cancer*, **53**, 542–546

Gresham, G.A. and Rue, III L.W. (1985) Squamous cell carcinoma of the liver. *Hum.Pathol.*, **16**, 413–416

Pawelczak, I., Pichurski, R., Sandlak, M. and Konecki, J. (1987) Pierwotny rak plaskonablonkowy watroby. *Wiad Lek*, **40**, 188–190

Lynch, M.J., McLeod, M.K., Weatherbee, L., Gilsdorf, J.R., Guice, K.S. and Eckhauser, F.E. (1988) Squamous cell cancer of the liver arising from a solitary benign nonparasitic hepatic cyst. *Am.J.Gastroenterol.*, **83**, 426–431

Mandai, K., Moriwaki, S., Doihara, H. *et al.* (1989) Adenosquamous carcinoma and squamous cell carcinoma of the liver: a resected case and three autopsied cases. *Gan No Rinsho*, **35**, 1439–1447

Flagg, R.S. and Robinson, D.W. (1967) Solitary nonparasitic hepatic cysts. Report of oldest known case and review of the literature. *Arch.Surg.*, **95**, 964–973

Sanfelippo, P.M., Beahrs, O.H. and Weiland, L.H. (1974) Cystic disease of the liver. *Ann.Surg.*, **179**, 922–925

Richmond, H.G. (1956) Carcinoma arising in congenital cysts of the liver. *J.Pathol.Bacteriol.*, **72**, 681–683

Ameriks, J., Appleman, H. and Frey, C. (1972) Malignant nonparasitic cyst of the liver: case report. *Ann.Surg.*, **176**, 713–717
Lloyd Jones, W., Mountain, J.C. and Warren, K.W. (1974) Symptomatic non-parasitic cysts of the liver. *Br.J.Surg.*, **61**, 118–123
Moore, S., Gold, R.P., Lebwohl, O., Price, J.B. and Lefkowitch, J.H. (1984) Adenosquamous carcinoma of the liver arising in biliary cystadenocarcinoma: clinical, radiological, and pathologic features with a review of the literature. *J.Clin.Gastroenterol.*, **6**, 267–275

*(Accepted by S.Bengmark 25 September 1991)*

